# City of London Medical Chirurgical Society

**Published:** 1831-01-01

**Authors:** 


### XXIV.

CITY OF LONDON MEDICAL AND CHIRURGICAL SOCIETY. Established May 1830.

RULES.—1st. That the above be the name and title of this Society, and that it consist of six official Members, four of whom shall form a Quorum.

2d. That one of the six Members shall deliver a Lecture in his turn at his own house, or appoint a proxy, on every Wednesday throughout the year, until two courses are completed.

3d. That the Members shall assemble at the Lecturer's house at 6 o'clock p. m. The Lecture to commence at half-past six, at which time every absent Member shall be fined 2s. 6d.—the Lecturer, if absent, 5s. The non-payment of the fines shall subject the party to exclusion.

4th. That a Member shall be allowed to invite medical or non-medical friends on the evening of his Lecture only.

5th. That a proportionate and specific time be allowed by the Secretary to each Member for reading the various publications, at the expiration of which time they shall be forwarded in order from one Member to another.

6th. That no expences be incurred, nor business transacted, but by a majority.

7th. That a report of the proceedings of this Society be published from and after the expiration of the first year of its establishment.

8th. That a President, Secretary, Treasurer, and Librarian, be balloted for annually, who shall report upon the state of the Society every three months.

9th. That at each weekly meeting, a Chairman be appointed for the evening, in the absence of the President.

10th. That each Member shall subscribe every four weeks towards the funds of the Society; which shall be applied to the purchase of Instruments, Medical and Chirurgical Publications, &c., for the use of the Members only.

11th. That new Members may be unanimously elected into the Society after having been proposed, three weeks' notice of which shall be given to all the Members. The said Members, so elected, not to fill any office or situation in the Society till the expiration of one year after their admission, except in cases of death, resignation, or exclusion, of Members in office.

12th. That if any Member shall act offensively or injuriously towards another Member, he shall be called upon for an apology. The refusal of which shall subject him to exclusion.

13th. That Bye Laws be made by a majority of Members, and recorded by the Secretary.

14th. That each Member on admission sign the above Rules.

